# Catalytic properties, functional attributes and industrial applications of β-glucosidases

**DOI:** 10.1007/s13205-015-0328-z

**Published:** 2015-12-31

**Authors:** Gopal Singh, A. K. Verma, Vinod Kumar

**Affiliations:** 1 Institute of Himalayan Bioresource Technology, Palampur, 176062 India; 2 Department of Biochemistry, College of Basic Sciences and Humanities, G. B. Pant University of Agriculture and Technology, Pantnagar, 263145 India; 3 Department of Biotechnology, Akal College of Agriculture, Eternal University, Baru Sahib, Sirmour, 173101 India

**Keywords:** β-Glucosidases, Glycoside hydrolase, Cellulosome, Glucosides, Cellulase

## Abstract

β-Glucosidases are diverse group of enzymes with great functional importance to biological systems. These are grouped in multiple glycoside hydrolase families based on their catalytic and sequence characteristics. Most studies carried out on β-glucosidases are focused on their industrial applications rather than their endogenous function in the target organisms. β-Glucosidases performed many functions in bacteria as they are components of large complexes called cellulosomes and are responsible for the hydrolysis of short chain oligosaccharides and cellobiose. In plants, β-glucosidases are involved in processes like formation of required intermediates for cell wall lignification, degradation of endosperm’s cell wall during germination and in plant defense against biotic stresses. Mammalian β-glucosidases are thought to play roles in metabolism of glycolipids and dietary glucosides, and signaling functions. These enzymes have diverse biotechnological applications in food, surfactant, biofuel, and agricultural industries. The search for novel and improved β-glucosidase is still continued to fulfills demand of an industrially suitable enzyme. In this review, a comprehensive overview on detailed functional roles of β-glucosidases in different organisms, their industrial applications, and recent cloning and expression studies with biochemical characterization of such enzymes is presented for the better understanding and efficient use of diverse β-glucosidases.

## Introduction

**Fig. 1:**
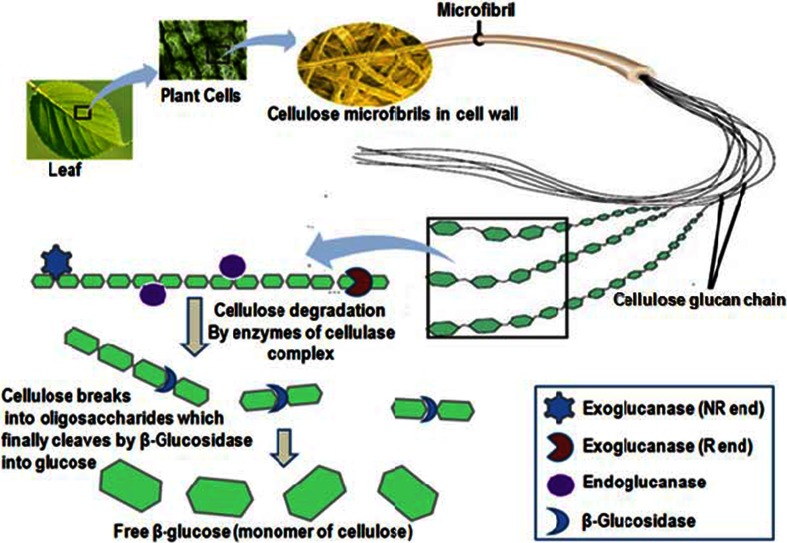
Diagrammatic overview of cellulose metabolism by cellulase system: during cellulose hydrolysis, cellulase along with exo and endoglucanase acts on the cellulosic fibers and hydrolyzed it into the smaller sized oligosaccharides. These smaller molecules are finally utilized by β-glucosidase as a substrate to release glucose as the final product of complete hydrolysis of cellulosic substance

β-Glucosidases (β-d-glucopyrranoside glucohydrolase) [E.C.3.2.1.21] are the enzymes which hydrolyze the glycosidic bond of a carbohydrate moiety to release nonreducing terminal glycosyl residues, glycoside and oligosaccharides (Bhatia et al. 2002; Morant et al. 2008; Cairns and Esen 2010; Li et al. 2013). These enzymes are present in all kinds of organisms including bacteria, archaea, and eukaryotes, and play several important roles such as biomass conversion in microorganisms, breakdown of glycolipids and process of lignification, involve in defense against pests, phytohormones activation, catabolism of cell wall in plants and both plant–microbes and plant–insects interaction. β-Glucosidase also plays an important role in the treatment of Gaucher’s disease (resulting from a deficiency of β-glucosidase) in which accumulation of glycoceramides takes place in the lysosomal tissues (Butters 2007). β-Glucosidases are the essential part of cellulase system (cellulose metabolizing enzymes) and catalyze the last and final step in cellulose hydrolysis. Cellulase enzymes hydrolyze the cellulose to produce cellobiose and other short oligosaccharides which are finally hydrolyzed to glucose by β-glucosidase. All the enzymes, which are involved in cellulose hydrolysis, are normally grouped as cellulase system (Fig. 1). It consists of the following enzymes: endoglucanase (endo-1, 4–β-glucanase [E.C.3.2.1.4]), exoglucanase (cellobiohydrolase) (exo-1, 4-β-glucanase [E.C.3.2.1.91]) and β-glucosidase (β-d-glucoside glycohydrolase [E.C.3.2.1.21]) (Teeri 1997). The endoglucanase randomly hydrolyzes the β–1–4 bonds in the middle portion of cellulose molecule and the exoglucanase acts at reducing and non-reducing ends to release the cellobiose and other oligosaccharides. Finally, these oligosaccharides are converted to glucose by β-glucosidase (Bhat and Bhat 1997).

β-Glucosidases are widely used in the various biotechnological processes, including the production of biofuel and ethanol from cellulosic agricultural wastes and synthesis of useful β-glucosides (Li et al. 2013). These enzymes are employed in industry for hydrolysis of bitter compounds during juice extraction and liberation of aroma from wine grapes (Gueguen et al. 1998; Harhangi et al. 2002). In flavor industry, β-glucosidases are the key enzymes in the enzymatic release of aromatic compounds from glucosidic precursors present in fruits and fermenting products (Krisch et al. 2010). Also, this has large potential for application in food processing industries and used as a flavor enzyme to enhance the flavor of wine, tea and fruit juice (Keerti et al. 2014). β-Glucosidases play an important role in flavor liberation from glucosylated (β-glucosides conjugated) precursors in fruits and other plant tissues (Krisch et al. 2010). Cleavage of phenolic and phytoestrogen glucosides from fruits and vegetables is also carried out by applying this enzyme to extract medicinally important compounds and to enhance the quality of beverages (Schroder et al. 2014). β-Glucosidases hydrolyzed anthocyanine products, i.e., anthocyanidins and sugar aglycones are less soluble than anthocyanines, possess little colour, tend to precipitate and can be removed more easily. β-Glucosidases can improve the organoleptic properties of citrus fruits and juices in which bitterness is in part due to a glucosidic compound, naringin whose hydrolysis requires in succession, an α-rhamnosidase and a β-glucosidase (Riou et al. 1998). β-Glucosidases have been the subject of recent research due to the key role of these enzymes in biological processes and for many biotechnological applications. In present review, we briefly explain the generalized action mechanisms of β-glucosidase enzymes, their functional role in different types of organisms and significant contribution in different industries along with a brief look on the current research for improving the efficiency of this industrially important enzyme.

## Types of β-glucosidases and their classification

β-Glucosidases are common among plants, fungi and bacteria, and showed an identical similarity with respect to their sequences and structures. They can be classified on the basis of their substrate activity or their nucleotide sequence identity. Based on substrate specificity, β-glucosidases are grouped into three classes: (i) aryl-β-D-glucosidases (having strong affinity for aryl-β-D-glucosides), (ii) cellobiases (hydrolyze only disaccharides) and (iii) broad specificity glucosidases (exhibit activity on many substrate types and are the most commonly found β-glucosidases) (Rajan et al. 2004). On the basis of sequence homology, β-glucosidases have been divided into two sub-families (i) BGA (β-glucosidases and phospho-β-glucosidases from bacteria to mammals) and (ii) BGB (β-glucosidases from yeasts, molds and rumen bacteria) (Cantarel et al. 2009; Krisch et al. 2010). An alternative classification system for glycoside hydrolases based on amino acid sequence and structural similarity has also been developed (Henrissat and Davies 1997). In this system, those enzymes with overall amino acid sequence similarity and well-conserved sequence motifs are considered in a single family. At present, 133 glycoside hydrolase (GH) families are listed in the frequently updated Carbohydrate Active enZYme (CAZY) website (http://www.cazy.org) (Cantarel et al. 2009; Cairns and Esen 2010). These families are further classified into clans. The families with similar catalytic domain structures and conserved catalytic amino acids, suggestive of a common ancestry and catalytic mechanism, are grouped under the same clan. Clan GH-A contains largest number of families including β-glucosidase containing families GH1, GH5 and GH30. Largest number of characterized β-glucosidases belongs to GH1 family. Family GH1 includes β-glucosidases from archaebacteria, plants and mammals, and family GH3 comprises β-glucosidases of some bacterial, mold and yeast origin (Cairns and Esen 2010; Krisch et al. 2010).

International Union of Biochemistry and Molecular Biology (IUBMB) classified GH families into the structurally determined families (Henrissat and Davies 1997; Harris et al. 2010). This family classification based on the structural features of the enzymes is more informative than substrate specificity because the complete range of substrates is very difficult to determine for individual enzymes. The structural features of a family can be used to study the structures of other members of the same family by applying some bioinformatics tools and system biology approach. Tertiary structure, particularly at the active site, dictates the enzyme mechanism and their substrate specificity. The family classification also explains the evolution of the glycoside hydrolases (Lynd et al. 2002). A classical (β/α)_8_ barrel Folds as a key feature of GH1 family β-glucosidase was reported in X-ray crystallographic structure of β-glucosidase BGL1A from a basidiomycete (*Phanerochaete chrysosporium*) by Nijikken et al. (2007). Structure of human cytosolic β-glucosidase was also illustrated by X-ray crystallography and reported the existence of the same (β/α)_8_ barrel (Tribolo et al. 2007).

## Catalytic mechanism of β-glucosidases

**Fig. 2:**
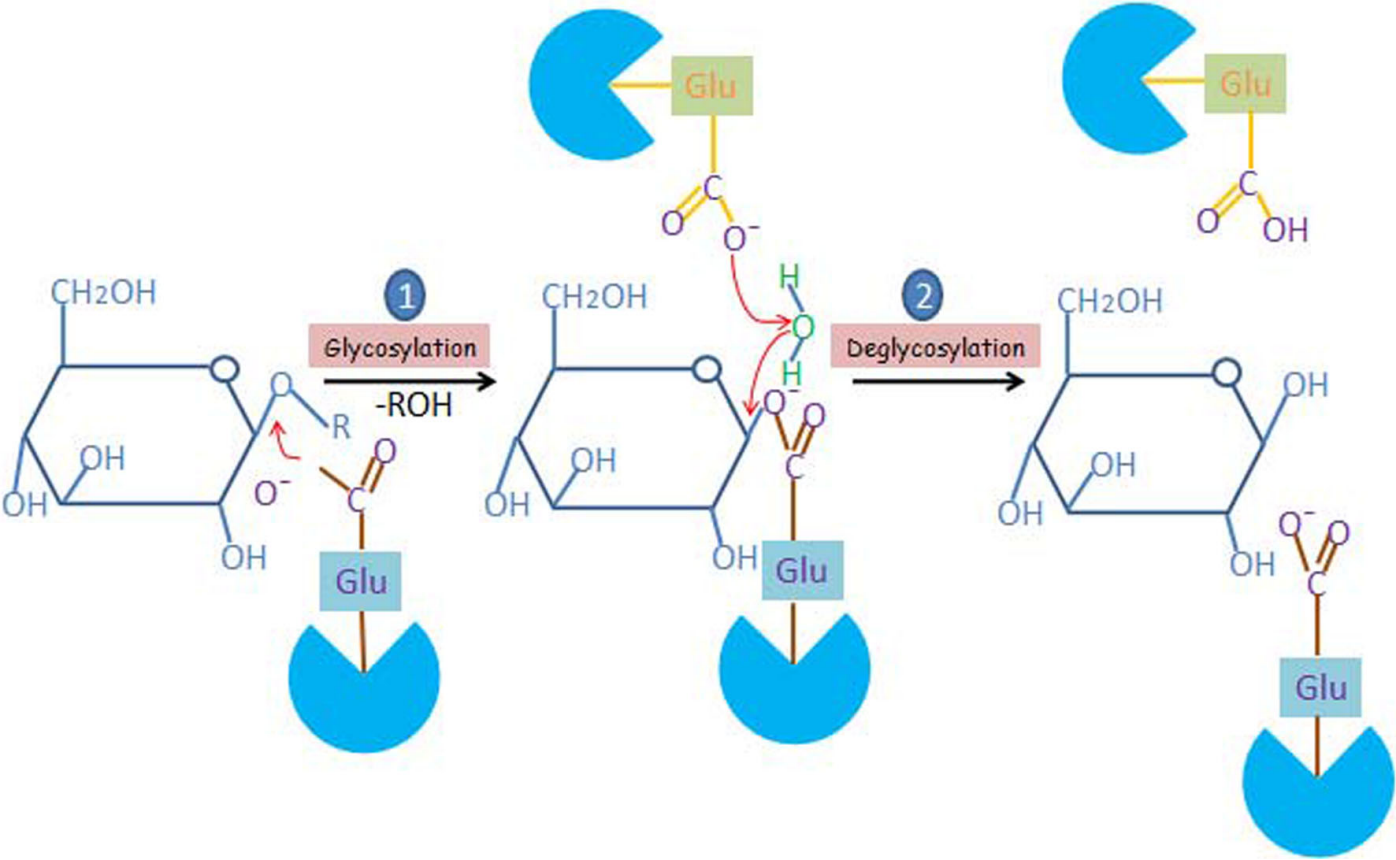
Proposed “retaining” mechanism for hydrolysis of β-glycosidic bond by β-glucosidase: (1) during first glycosylation step, a conserved glutamate residue acts as nucleophile and attacks on the glycosidic bonds or cellobiose and other oligosaccharides formed by the hydrolytic action of other enzyme of cellulase system. This results into the formation of an enzyme-substrate intermediate complex, (2) during second step called deglycosylation, an another conserved glutamate residue activates a water molecule present in the proximity by general acid/base catalyst reaction and now this activated water molecule acts on the intermediate complex to release the free glucose residue

For elucidating the catalytic mechanism of the enzyme and the active site topology, several techniques such as pH-dependence, inhibition, isotopic effect, and structure–reactivity studies (Kempton and Withers 1992), essential amino acid labeling with fluorosugars (Withers et al. 1992), reactions with deoxy substrate analogues (Street et al. 1992), and site-directed mutagenesis (Wang et al. 1995) have been used. β-Glucosidases cleave β-glycosidic bonds between two or more carbohydrates, or between a carbohydrate and a non-carbohydrate moiety (http://www.cazy.org/Glycoside-Hydrolases.html). Most β-glucosidases that have been characterized (i.e., GH1, GH3 and GH30 family enzymes) are retaining enzymes, and they perform catalysis in two steps, glycosylation and deglycosylation. Their catalytic mechanism is described diagrammatically in Fig. 2. Glutamate is the key active site residue and conserved among all reported β-glucosidases (Davies and Henrissat 1995; Wang et al. 1995). Two glutamate residues performed the overall catalytic reaction of β-glucosidase and one of them acts as a nucleophile (conserved as ‘I/VTENG’ motif) and the second residue works as a general acid/base catalyst (conserved as a ‘TFNEP’ motif) (Davies and Henrissat 1995). In the initial (glycosylation) step, glutamate, which acts as nucleophile, undergoes a nucleophilic attack on the anomeric carbon and results in a glucose–enzyme intermediate product. In the second (deglycosylation) step, a water molecule, which is activated by acid/base catalyst glutamate residue, acts as a nucleophile and breaks the glycosidic bond to release glucose (Litzinger et al. 2010). The formation of the covalent intermediate was first demonstrated with the GH1 β-glucosidase from *Agrobacterium* sp. by covalent labeling with 2, 4-dinitrophenyl-2-deoxy-2-fluoroglucoside (Withers et al. 1987, 1990). Tribolo et al. (2007) have reported X-ray crystallographic structure of human cytosolic β-glucosidase with the pocket shaped active site containing two glutamate residues and formed by large surface loops, surrounding the C termini of the barrel of β-strands. Out of two catalytic glutamate residues, the acid/base catalyzing residue was located on strand 4 while the nucleophilic residue was located on strand 7.

β-Glucosidases from GH9 family use an inverting mechanism, in which an activated water molecule makes a direct nucleophilic attack on the anomeric carbon to displace the aglycone in a single step (Qi et al. 2008). The catalytic base extracts a proton from the incoming water molecule while the catalytic acid protonates the leaving group aglycone.

## Functional roles of β-glucosidases in different organisms

Enzymes are the biocatalysts and involved in almost all biological processes. β-Glucosidases are kind of enzymes which regulate both synthesis as well as breakdown process and involved in many essential bioconversions in all kinds of life forms. The roles of β-glucosidases in different organisms are discussed briefly in following sections.

### In microorganisms

Microorganisms are one of the simplest and primitive life systems evolved in our planet. Many studies have been carried out on microorganisms with respect to β-glucosidases but most of them are focused on their industrial application rather than their endogenous function in the target microorganisms. β-Glucosidases are reported in all forms of microorganisms such as bacteria (Isorna et al. 2007; Chen et al. 2010a; Verma et al. 2013), archea (Park et al. 2007; Cobucci-Ponzano et al. 2010; Li et al. 2013; Schroder et al. 2014) and fungi (Krisch et al. 2010; Sorensen et al. 2013). Archaea are considered as best source for industrially suitable enzymes due to their functional stability in higher temperature which is useful for their utilization in industrial operations (Li et al. 2013). These enzymes play important roles in some fundamental biological processes such as in degradation of cellulose and other carbohydrates for nutrient up-take, cell wall mechanism, host–pathogen, and symbiotic association. β-Glucosidases enable phytopathogenic fungi to colonize their host plant tissues by hydrolyzing toxic glucosides to less toxic or less soluble aglycone (Collins et al. 2007). Bacterial β-glucosidases are components of large complexes called cellulosomes, contain polysaccharide degrading endoglucanases and carbohydrate binding proteins to localize the complex and to the cellulose surface and the cell membrane. In bacteria and fungi, β-glucosidases are mainly a part of the cellulase enzyme system and are responsible for the hydrolysis of short chain oligosaccharides and cellobiose (resulting from the synergistic action of endoglucanases and cellobiohydrolases) into glucose in a rate-limiting step (Isorna et al. 2007). It was found that the enzyme activity of cellulose-degrading enzymes decreases as the glucose chain length increases (Bhatia et al. 2002).

The principal role of β-glucosidase in cellulolytic microorganisms is to catalyze the hydrolysis of cellobiose and cello-oligosaccharides, producing glucose during bioconversion. These soluble substrates of β-glucosidase are produced from insoluble cellulose by the action of other members of the cellulose system of enzymes (Doi and Kosugi 2004). β-Glucosidase is a useful enzyme from soil microbes and contributes in maintenances of soil quality because of its central role in soil organic matter cycling (Turner et al. 2002). They are also involved in host–pathogen and symbiotic relationships as they catalyze the breaking through plant cell walls to establish pathogenic or symbiotic relationships (Gilbert et al. 2008).

### In plants

In plants, β-glucosidases performed many functions. Morant et al. (2008) explain the involvement of β-glucosidases in plant metabolisms. These are involved in many key processes such as formation of required intermediates for cell wall lignification (Escamilla-Trevino et al. 2006), degradation of endosperm’s cell wall during germination (Leah et al. 1995), in indole alkaloid biosynthesis (Barleben et al. 2005) activation of plant hormones (Lee et al. 2006; Verma et al. 2011), Cyanogenesis (release of toxic cyanide) (Zagrobelny et al. 2004) and in plant defense against biotic stresses (Jones et al. 2000). Plants contain defense glycosides in non-active form which are activated by the action of β-glucosidase enzymes and released as toxic compounds (Jones and Vogt 2001; Knudsen 2014). In general, the defense glycosides are stored in a different cell or a different cellular compartment from the β-glucosidases that hydrolyze them to release toxic compounds. The defense compounds tend to be stored in the vacuole, while their corresponding β-glucosidases are often found in the apoplast or plastid (Zagrobelny et al. 2008). Verdoucq et al. (2004) studied a plant β-glucosidase which plays important role in plant defense against pests. These enzymes cleave specific β-glucosides to release toxic aglycone moieties. In *Lotus japonicas,* β-glucosidase catalyzes the bioactivation of hydroxynitrile glucosides which provide the strength against herbivores and pathogens (Morant et al. 2008; Knudsen 2014). The family 1 β-glucosidase enzyme of *Sorghum biocolor* (Dhr1) has shown a strict specificity for its natural substrate dhurrin. While, maize β-glucosidase (Glu1), which shares 70 % sequence identity with Dhr1, hydrolase a broad spectrum of substrate in addition to its natural substrate 2-*O*-β-glucopyranosyl-4-hydroxy-7-methoxy-1,4-benzoxaxin-3-one. Sherameti et al. (2008) observed that β-glucosidase-mediated defenses are also required for endophytic fungi to develop symbiotic relationships with plants, evidently by modulating the growth of these microorganisms. β-Glucosidases appear to play roles in both the degradation of oligosaccharides presented in cell wall and release of monolignols from their glycosides to allow lignification to stabilize secondary cell walls (Kuntothom et al. 2009). These enzymes also play essential roles in plant’s secondary metabolisms. The monoterpene alkaloid intermediate is hydrolyzed by a specific β-glucosidase to allow metabolism to various monoterpene alkaloids, depending on the plant (Barleben et al. 2007). β-Glucosidases could play metabolic roles to release glucosyl blocking groups from metabolic intermediates and allow metabolism to various natural products, many of which are of medicinal importance (Nomura et al. 2008).

### In mammals

Mammals contain several β-glucosidases, including the family GH1 lactase-phloridzin hydrolase and cytoplasmic β-glucosidase, the GH30 human acid β-glucosidase (GBA1) and the bile acid β-glucosidase or GBA2. These enzymes are thought to play roles in metabolism of glycolipids and dietary glucosides. In addition, a group of related family GH1 proteins is thought to play signaling functions. Currently, five GH1 proteins are known in humans: lactase-phloridzin hydrolase (LPH), cytoplasmic β-glucosidase, Klotho (α-Klotho, KL) β-Klotho (β-KL), and Klotho-LPH-related protein (KLPH). LPH, an intestinal hydrolase involved in food digestion, has both β-glucosidase activity toward exogenous glucosides, such as phloridzin, and β-galactosidase activity toward lactose (Arribas et al. 2000). Perhaps the best-studied mammalian β-glucosidase is the human acid β-glucosidase, which is generally considered a glucosyl ceramidase. Defects in the function of this enzyme and its transport to the lysosome lead to Gaucher’s disease, in which glycoceramides accumulate in the lysosomes of tissue leukocytes leading to their swelling (Butters 2007). Mammalian cytosolic β-glucosidase from liver cells also has ability to hydrolyze many common dietary xenobiotics, including glycosides of phytoestrogens, flavonoids, simple phenolics and cyanogens, and prevents from their hazardous effect (Hays et al. 1998; Berrin et al. 2002). Such kind of cytosolic β-glucosidase was also reported from small intestine, spleen and kidney (Berrin et al. 2002). Takagi et al. (1999) reported a β-glucocerebrosidase, a kind of β-glucosidase in mammalian stratum corneum which has a role in lipid distribution during membrane structural maturation and epidermal homeostasis.

### In insects and other animals

The insect β-glucosidase has both merits and demerits: these are the main enzymes used for converting wood-cellulose to glucose for biofuel production and can also be an important part of the mechanism which leads to serious damage to wood structures, landscaping trees and many agricultural crops (Zhang et al. 2012). Due to these twofold implications, β-glucosidase from insect origin is a concern of several ongoing studies to enhance the cellulose degradation and development of cellulase-specific inhibitors as termiticides for insect and pest control (Yapi et al. 2009). For these purposes, a lot of insects such as silkworm (*Bombyx mori*) (Byeon et al. 2005), *Reticulitermes flaviceps *(Xue et al. 2008), palm weevil (*Rhynchophorus palmarum*) (Yapi et al. 2009), termite (*Reticulitermes santonensis)* (Matteotti et al. 2011), cockroach (*Periplaneta americana*) (Koffi et al. 2012), red palm weevil (*Rhynchophorus ferrugineus*) (Riseh et al. 2012) and higher termites (Bujang et al. 2014) have been studied for the isolation and characterization of an efficient β-glucosidase.

*Drosophila melanogaster* genome contains only one GH1 gene, suggesting that insects may not synthesize this gene family at an early stage, other insects have adapted glycosides and glycoside hydrolases from the plants on which they feed for protection and digestive purposes. These genes have since evolved to provide help for the unique needs of the herbivorous insects in their battle with plant defenses to utilize the plant nutrients. A few digestive β-glycosidases from GH1 have been isolated from insect larvae that feed on plant. The larval β-glycosidases can hydrolyze gluco-oligosaccharides and plant glycosides, such as cellobiose, gentiobiose and amygdalin (Marana et al. 2001). β-Glucosidase was also reported in the gut contents of the snail (*Achatina achatina*) (Umezurike 1976).

## Isolation, cloning and expression studies for production of efficient and novel β-glucosidase

To obtain an improved enzyme, researchers have adopted several approaches like heterogeneous expression of β-glucosidase genes from different sources (Byeon et al. 2005; Chan et al. 2011; Aftab et al. 2012), characterization of immobilized enzyme in several agents (Keerti et al. 2014) and side-directed mutagenesis to enhance the efficacy of enzymes (Wang et al. 1995; Liu et al. 2011; Agrawal et al. 2012). Cloning and expression in suitable host for bulk production of β-glucosidase enzyme is one of the primitive concerns about ongoing research on β-glucosidase. Cloned enzyme also exhibits some advantages such as showing high isoflavone glucoside-hydrolyzing activity (Kuo and Lee 2007), more resistant to glucose inhibition (Jabbour et al. 2012), and more efficient for solid state fermentation (Krisch et al. 2010). Several β-glucosidases have been cloned from bacteria, yeast, fungi, plant and animal sources with the intent of producing this enzyme on a large scale for various biotechnological applications (Collins et al. 2007; Chang et al. 2011; Aftab et al. 2012). For expression of β-glucosidase, different kind of host organisms including *Pichia pastoris*, Trichoderma *reesei* and *E. coli* species were used (Harhangi et al. 2002; Murray et al. 2004; Chang et al. 2011). For β-glucosidase gene cloning studies, following three strategies have been used: first, formation of a genomic DNA library followed by selection of the clones by screening for β-glucosidase production; Second, starting with a cDNA library (or a genomic library), screening of recombinant clones by specific nucleotide probes designed from a knowledge of the polypeptide sequence; and third strategy is primer designing of existing gene sequence and PCR based cloning of gene (Bhatia et al. 2002). A detailed account of properties of heterogeneously expressed β-glucosidases from various bacteria, fungi, yeasts, and plant sources is given in Table 1.

**Table 1:** Characteristics of heterogeneously expressed β-glucosidases isolated from different types of organisms

Type of organism	Source organism	Expression host	Mol. Mass (kDa)	No. of Amino acids	pH optima	Temp. optima (°C)	GH family	References
Bacteria	*Acetobacter xylinum*	*E. coli*	79	739	–	–	GH3	Tajima et al. (2001)
	*Vibrio cholera*	*E. coli*	65	574	6.0–6.5	37–42	GH9	Park et al. (2002)
	*Thermus flavus*	*E. coli*	49	431	5.0–6.0	80–90	GH1	Kang et al. (2005)
	*Thermus thermophiles*	*E. coli*	48.7	431	8.5	90	GH1	Gu et al. (2009)
	*Exiguobacterium oxidotolerans*	*E. coli*	51.6	448	7	35	GH1	Chen et al. (2010a)
	*Cellulomonas biazotea*	*E. coli*	48	447	–	–	GH1	Chan et al. (2011)
	*Exiguobacterium* sp.	*E. coli*	52	450	7.0	45	GH1	Chang et al. (2011)
	*Micrococcus antarcticus*	*E. coli*	48	472	6.5	25	GH1	Fan et al. (2011b)
	*Sphingomonas* sp.	*E. coli*	49.3	447	5.0	37	GH1	Wang et al. (2011)
	*Bacillus licheniformis*	*E. coli*	53	466	6	50	GH1	Zahoor et al. (2011)
	*Bacillus licheniformis*	*E. coli*	52.2	–	6.0	60	GH16	Aftab et al. (2012)
	*Fervidobacterium islandicum*	*E. coli*	53.41	459	6.0–7.0	90	GH1	Jabbour et al. (2012)
Yeast	*Candida wickerhamii*	*E. coli*	72	–	7	37–40	GH1	Skory and Freer (1995)
	*Pichia etchellsii*	*E. coli*	50	–	6.5–7.0	50	–	Pandey and Mishra (1997)
	*Pichia etchellsii*	*E. coli*	52.1	504	7–9	45	GH1	Roy et al. (2005)
Other Fungi	*Piromyces* sp.	*Pichia pastoris*	75.8	–	6.0	39	GH1	Harhangi et al. (2002)
	*Uromyces fabae*	–	92.4	843	–	–	GH3	Haerter and Voegele (2004)
	*Sclerotinia sclerotiorum*	–		–	5.0	55–60	–	Issam et al. (2004)
	*Talalaromyces emersonii*	*Trichoderma reesei*	90.59	857	4.02	71.5	GH3	Murray et al. (2004)
	*Thermoascus aurantiacus*	*Pichia pastoris*	–	–	5.0	70	GH3	Hong et al. (2007)
	*Aspergillus fumigatus*	*Pichia pastoris*	91.47	–	6.0	60	GH3	Liu et al. (2012a)
	*Neosartorya fischeri*	*E. coli*	–	529	6.0	40	GH1	Ramachandrana et al. (2012)
Animal/insects	Human liver	*Pichia pastoris*	53	496	6.5	50	GH1	Berrin et al. (2002)
	*Bombyx mori*		57	491	6.0	35	GH1	Byeon et al. (2005)
	*Reticulitermes santonensis*	*E. coli*	–	–	6.0	40	GH1	Matteotti et al. (2011)
	*Macrotermes barneyi*	*E. coli*	54	493	5.0	50	GH1	Wu et al. (2012)
	*Miscanthus sinensis*	*E. coli*	85	779	5.0	38	GH3	Li et al. (2014)
Plant	*Pinus contorta*	*E. coli*	–	513	–	–	GH1	Dharmawardhana et al. (1999)
	*Rauvolfia serpentine*	*E. coli*	61	540	5.0	28	GH1	Warzecha et al. (2000)
	*Glycine max*	*E. coli*	58		7.0	30	GH1	Suzuki et al. (2006)

## Industrial application of β-glucosidases

β-Glucosidases have both cleavage and synthesis activity of glycosidic bonds and thus involved in many crucial biological pathways such as cellular signaling, biosynthesis and degradation of structural and storage polysaccharides, host–pathogen interactions, as well as in a number of biotechnological applications. Recently, the increased demand of energy has strongly stimulated the research on the conversion of lignocellulosic biomass into reducing sugars for the subsequent ethanol production. β-Glucosidases have been the focus because of their important roles in a variety of fundamental biological processes and the synthesis of useful β-glucosides. Although the β-glucosidases of different sources have been investigated, the amounts of β-glucosidases are insufficient for effective conversion of cellulose.

The cellulolytic fungal β-glucosidases have also been the subject of numerous investigations by various research groups (Murray et al. 2004; Liu et al. 2012a). The fungal enzymes are used in several biotechnological processes, including development of novel carbohydrate foods, alcohol-based fuels and other commercial products from cellulose (Krisch et al. 2010). Liu et al. (2012a) isolated a thermostable native β-glucosidase from the lignocellulose-decomposing fungus *Aspergillus fumigatus* Z5. Furthermore, when it was added to lignocellulosic materials, the release of phenolic compounds increased, indicating that cellulose-degrading enzymes may also be involved in the breakdown of polymeric phenolic compounds.

### β-Glucosidase in ethanol and biofuel production

The increased energy consumption demand and the depletion of fossil resources have laid the foundation for a shift towards sustainable production of biofuels (Sorensen et al. 2013). The use of plant biomass in the form of dedicated energy crops or cellulosic agricultural waste as an abundant and inexpensive material for biofuel is one of the current focus of industrial as well as research aspect. Most bioconversion is focused on the production of a sugar platform of simple sugars which can then biologically or chemically be converted into fuels (e.g., ethanol, butanol and hydrocarbons) (Cherubini 2010). Recently, Li et al. (2014) cloned and characterized a β-glucosidase from rumen microbes of cattle feeding with *Miscanthus sinensis* and this plant is used as an ideal source of biofuel. Coating of β-glucosidase on polymer nanofibers was also found more efficient for cellulosic ethanol production (Lee et al. 2010). β-Glucosidases as part of the cellulase enzyme complex hydrolyze cellobiose and cello-oligosaccharides to yield glucose which is fermentable by yeasts into fuel ethanol. Ethanol production from cellulose is performed via the degradation of cellulose to cello-oligosaccharides and glucose, followed by the conversion of glucose to ethanol by microorganisms (Kotaka et al. 2008). The cellulose is degraded by endoglucanases and exoglucanases, producing cellobiose and some cello-oligosaccharides, which can be converted to glucose by β-glucosidase. The reaction catalyzed by β-glucosidase is the most important step in the degradation of cellulose because it limits the efficiency of hydrolysis and could relieve the cellobiose-mediated inhibition of exoglucanase and endoglucanase (Zhenming et al. 2009). Liu et al. (2012b) reported a yeast strain *Clavispora* NRRL Y-50464 that is able to utilize cellobiose as sole source of carbon and produce β-glucosidase enzyme activity for cellulosic ethanol production. It was also observed that this yeast was tolerant to the major inhibitors derived from lignocellulosic biomass pre-treatment such as 2-furaldehyde (furfural) and 5-(hydroxymethyl)-2-furaldehyde (HMF), and converted furfural into furan methanol in less than 12 h and HMF into furan-2,5-dimethanol within 24 h in the presence of 15 mM each of furfural and HMF. The ethanol production of 23 g/l was obtained without addition of exogenous β-glucosidase by this strain. However, the most widely used cellulase from *Trichoderma viridae* has a poor β-glucosidase activity and the accumulation of cellobiose will lead to product inhibition. Addition of thermo-tolerant β-glucosidases to commercial cellulase enzyme preparations resulted in synergistic effect and increased reducing sugar concentration (Krisch et al. 2010).

Ethanol producing bacteria have attracted much attention because their growth rate is higher than that of the *Saccharomyces cerevisiae* which is normally used for commercial production of fuel alcohol and to make industrial ethanol production more economical. Yanase et al. (2005) carried out his research work on *Zymomonas mobilis,* an ethanol producing bacterium. It has been observed that *Zymomonas mobilis* showed high growth rate and high specific ethanol production, which makes it an attractive candidate for industrial ethanol production, but its narrow spectrum of fermentable carbohydrates reduced its use for fuel ethanol production. To overcome from this limitation, genetic manipulation in β-glucosidase gene was used to expand the range of its carbohydrate substrates which lead to production of 0.49 g ethanol/g cellobiose by recombinant strain. Some of the recent studies focused on the use of microbial β-glucosidase for biofuel production from cellulosic waste are listed in Table 2.

**Table 2:** Recent studies on the isolation, cloning and characterization of β-glucosidase for ethanol production from cellulosic materials

Microorganism type	Name of organisms	Total ethanol production	References
Bacteria	*Exiguobacterium oxidotolerans*	–	Chen et al. (2010a)
	*Penicillium decumbens*	–	Chen et al. (2010b)
	*Cellulomonas biazotea*	–	Chan et al. (2011)
	*Clostridium phytofermentas*	25 mM	Tolonen et al. (2011)
	*Clostridium thermocellum*	1.80 g/l	Kim et al. (2013)
Yeast	*Saccharomycopsis fibuligera*	9.15 g/l	Jeon et al. (2009a)
	*Saccharomycopsis fibuligera*	–	Jeon et al. (2009b)
	*Issatchenkia orientalis*	29 g/l	Kitagawa et al. (2010)
	*Saccharomyces cerevisiae*	45 g/l	Ha et al. (2011)
	*Clavispora* NRRL Y-50464	23 g/l	Liu et al. (2012b)
	*Saccharomyces cerevisiae*	8.5 g/l	Tang et al. (2013)
Other Fungi	*Aspergillus oryzae*	21.6 g/l	Kotaka et al. (2008)
	*Aspergillus niger* and *Trichoderma reesei*	–	Chauve et al. (2010)
	*Penicillium decumbens*	–	Ma et al. (2011)
	*Agaricus arvensis*	–	Singh et al. (2011)
	*Neocallimastix patriciarum*	–	Chen et al. (2012)
	*Periconia* sp.	–	Dashtban and Qin (2012)
	*Penicillium simplicissimum* H-11	–	Bai et al. (2013)
	White rot fungi	–	Mfombep et al. (2013)
	*Acremonium thermophilum* and *Thermoascus aurantiacus*	–	Teugjas and Valjamae (2013)
	*Aspergillus nidulans, Aspergillus fumigatus, and Neurospora crassa*	–	Bauer et al. (2006)

### β-Glucosidase in wine making, tea and other beverages

In wine making, β-glucosidases play a key role in the enzymatic release of aromatic compounds from glycosidic precursors present in fruit juices, musts and fermenting products for wine making (Sestelo et al. 2004; Keerti et al. 2014), tea (Su et al. 2010) and fruit juice (Fan et al. 2011a). The natural process by endogenous plant β-glucosidases is very time consuming. Supplementation with efficient external enzymes may enhance aroma release (Gueguen et al. 1998). Monoterpenols in grapes (e.g., linalol, geraniol, nerol, citronclol, α-terpineol and Iinalol oxide) are linked to diglucosides, which contribute to the flavor of wine (Gunata et al. 1994). The enzymatic hydrolysis of these compounds requires a sequential reaction, which produce monoglucosides. Subsequently, monoglucosides are hydrolyzed by the action of β-glucosidases. The addition of glucose-tolerant exogenous β-glucosidase isolated from fungi (e.g., *A. oryzae*) was shown to improve the hydrolysis of glucoconjugated aromatic compounds and enhance wine quality (Riou et al. 1998). Sestelo et al. (2004) isolated and characterized β-glucosidase from wine strain of *Lactobacillus plantarum* which also performs the lactic acid fermentation and culminated need of applying additional enzymes to release flavorous compounds. Recently, Gonzalez-Pombo et al. (2011) isolated and characterized an extracellular β-glucosidase from *Issatchenkia terricola* to study the efficiency of immobilized enzyme to enhance the aroma flavor of white Muscat wine. In tea beverage industries, use of β-glucosidase enhances the content of essential oils (Krisch et al. 2010). In one report, Su et al. (2010) used an immobilized β-glucosidase to increase the essential oil content from 6.79 to 20.69 %. In citrus fruit juices, the hydrolysis of naringenin by β-glucosidase found to be reducing the bitterness of the juices (Fan et al. 2011a).

### β-Glucosidase in soy-based foods

Soyabean is a part of many foods and drink products and soy contains many glucosidic isoflavones like daidzin, genistin and glycitin. However, in soy-based foods the isoflavones are mainly in the inactive form of glycosides which can be hydrolyzed by applying additional β-glucosidase enzyme to convert them into aglycones (daidzein, genistein and glycitein) (Hati et al. 2015). Aglycone forms of isoflavones exhibit higher biological activity than their glucosidic forms and also absorbed faster in higher amounts during digestion (Izumi et al. 2000). Isoflavones found in soybean exhibit phytoestrogenic properties and are useful in treatment and prevention of various diseases such as cardiovascular disease and osteoporosis (Rimbach et al. 2008), prostate cancer and breast cancer (Liggins et al. 2000), relieve in menopause symptoms (Levis et al. 2010), estrogenic and antioxidant activity (Liu et al. 2010). In soy milk, the aglycon content was increased significantly either by treatment with β-glucosidase or by fermentation with a β-glucosidase producing *Lactobacillus* strain (Marazza et al. 2009). In the food industry, the application of gellan, an exopolysaccharide produced by *Sphingomonas paucimobilis*, is very limited because of its high viscosity and low solubility. Therefore, hydrolytic activity of β-glucosidases may be useful in the production of low-viscosity gellan foods. β-Glucosidases are also associated with removal of bitterness from citrus fruit juices (Roitner et al. 1984).

### β-Glucosidase in flavor industry

β-Glucosidases are widely used in the flavor improvement industry. Many hundreds of different β-glucosidic flavor precursors found in plants, and their hydrolysis by β-glucosidases enhance the quality of the beverages and foods produced from them. β-Glucosidases are key enzymes in the release of aromatic compounds from glucosidic precursors present in fruits and fermentating products (Schroder et al. 2014). β-Glucosidases can also be used to improve the organoleptic properties of citrus fruit juices, in which it reduced the bitterness that is due to a glucosidic compound, naringin (4,5,7-trihydroxyflavanone-7-rhamnoglucoside) (Roitner et al. 1984; Keerti et al. 2014). After treatment of β-glucosidases, the reduction of fruit bitterness or gellan hydrolysis leads to the reduction in viscosity of fruit juice was also observed (Schroder et al. 2014). Recently, Keerti et al. (2014) have isolated and characterized a thermostable β-glucosidase from *Bacillus subtilis* and applied it to enhance the quality of sugarcane juice after immobilizing it into alginate beads.

### Other applications of β-glucosidase

β-Glucosidases are used for the synthesis of oligosaccharides and alkyl-glycosides (Bankova et al. 2006). Oligosaccharides can be used as therapeutic agents, diagnostic tools and growth promoting agent. They have important functions in biological systems including fertilization, embryogenesis and cell proliferation. Alkyl-glycosides are nonionic surfactants with high biodegradability and also have antimicrobial properties. Hence, they have potential application in pharmaceutical, chemical, cosmetic, food and detergent industries as these can be hydrolyzed by β-glucosidase (Bankova et al. 2006). Enzymes from the source plants or other sources may be added to foods and beverages before, during, or after processing to enhance food quality. Thus, β-glucosidases with desirable properties may be focused for plant breeding programs, tissue culture and recombinant technologies to increase their overproduction in transgenic microbial or plant hosts and their catalytic properties for flavor enhancement and stability.

Aside from flavor enhancement, foods, feeds and beverages may be improved nutritionally by release of vitamins, antioxidants and other beneficial compounds from their glycosides. Opassiri et al. (2004) studied that vitamin B_6_ (pyridoxine) can be released from pyridoxine glucoside by β-glucosidase in rice. Other vitamins are also found as glucosides in different plants and release of their aglycones may improve their nutritional availability. This enzyme is also able to hydrolyze anthocyanins producing anthocyanidins and sugars. The resulting aglycones process little color and are less soluble than anthocyanins tend to precipitate and can be removed more easily. It is very much helpful in orange industry as it helps in color changing during pasteurization. β-Glucosidase supplementation was beneficial for single-stomached animals such as pigs and chickens in which cellulose degradation was enhanced by this enzyme leading to better nutrient utilization (Zhang et al. 1996).

As evident from previous sections, the wide functional implications and industrial applications of β-glucosidases make it a promising target for studies related to its higher production, novel enzyme, better stability, etc. Although β-glucosidases are having tremendous industrial demand but a suitable industrial β-glucosidase fulfilling all the desired properties is still lacking and studies are continued in anticipation of a novel enzyme with such properties.

In conclusion, an understanding of in vivo functional roles of these enzymes, biochemical properties, existing applications and well characterized heterogeneous expression technology discussed in this review will help in improvement of these enzymes using enzyme engineering and more relevant applications could be emerged in near future.
